# Significant variations in the cervical cancer screening rate in China by individual‐level and geographical measures of socioeconomic status: a multilevel model analysis of a nationally representative survey dataset

**DOI:** 10.1002/cam4.1321

**Published:** 2018-03-24

**Authors:** Heling Bao, Lei Zhang, Limin Wang, Mei Zhang, Zhenping Zhao, Liwen Fang, Shu Cong, Maigeng Zhou, Linhong Wang

**Affiliations:** ^1^ National Center for Chronic and Non‐communicable Disease Control and Prevention Chinese Center for Disease Control and Prevention Beijing China; ^2^ Research Centre for Public Health Tsinghua University Beijing China; ^3^ Melbourne Sexual Health Centre Alfred Health Melbourne Australia; ^4^ Central Clinical School Faculty of Medicine, Nursing and Health Sciences Monash University Melbourne Australia; ^5^ School of Public Health and Preventive Medicine Faculty of Medicine, Nursing and Health Sciences Monash University Melbourne Australia

**Keywords:** Mass screening, socioeconomic status, surveillance, uterine cervical neoplasms

## Abstract

Variations in cervical cancer screening rates in China have rarely been studied in depth. This study aimed to investigate cervical cancer screening rates in relation to both individual‐level and geographical measures of socioeconomic status (SES). Data were obtained from women aged 21 years or older by face‐to‐face interviews between August 2013 and July 2014 as part of the Chinese Chronic Diseases and Risk Factors Surveillance. The geographical variables were obtained from the 2010 Chinese population census. The cervical cancer screening rates and 95% confidence interval (CI) were estimated and mapped. Multilevel logistic regression models were fitted. Overall, only 21.4% (95% CI: 19.6–23.1%) of 91,816 women aged ≥21 years reported having ever been screened for cervical cancer and significant geographical variations at both province and county levels were identified (*P* < 0.01). The cervical cancer screening rates were the lowest among the poor [13.9% (95% CI: 12.1–15.7%)], uninsured [14.4% (95% CI: 10.3–18.4%)], less‐educated [16.0% (95% CI: 14.3–17.6%)], and agricultural employment [18.1% (95% CI: 15.8–20.4%)] women along with those residing in areas of low economic status [15.0% (95% CI: 11.8–18.2%)], of low urbanization [15.6% (95% CI: 13.4–17.7%)], and of low education status [16.0% (95% CI: 14.0–18.1%)]. The multilevel analysis also indicated that women with lower individual‐level measures of SES residing in areas with low geographical measures of SES were significantly less likely to receive cervical cancer screening (*P* < 0.0001). Despite the launch of an organized cancer screening program in China, cervical cancer screening rates remain alarmingly low and significant variations based on geographical regions and measures of SES still exist. It is therefore essential to adopt strategies to better direct limited available public resources to priority groups.

## Introduction

Population‐based screening rates largely determine the effectiveness of cervical cancer screening in reducing associated incidence and death rates 1. The proportion of eligible women screened for cervical cancer at least once or more often should be monitored in surveillance frameworks for noncommunicable diseases 2. Although nearly 90% of cervical cancer burden worldwide occurs in developing countries 3, significantly lower screening rates are usually reported among women in these countries than those in developed countries 4. Due to differences in the resources available based on the setting, the access to care, quality of care, and diagnosis for cervical cancer strikingly differ between developing and developed countries 4. Hence, identifying priority populations and achieving high screening rates for these target groups are urgently needed in a low‐resource setting 5.

A wide range of socioeconomic factors is associated with lower screening rates for cervical cancer. At the individual level, individuals with a low level of education and a lower health literacy 6, 7, who are uninsured and with a lower income 8, 9, or unemployed and with unhealthy lifestyles 10 are less likely to undergo cancer screening. At the geographical level, deprivation and urbanization 11, lack of well‐organized screening programs 12, insufficient healthcare workers, and low level of education 13, 14 are barriers to access to screening services. However, relatively few studies have examined the association of cervical cancer screening with both individual‐level and geographical measures of socioeconomic status (SES) at the same time 14.

In China, an estimated 28,010 women die from this preventable cancer, accounting for 12% of all cervical cancer deaths worldwide 15, 16. To date, a universal, well‐organized screening system for cervical cancer has not been established due to insufficient public health resources. In 2009, the Chinese government initiated the National Cervical Cancer Screening Program in Rural Areas (NACCSPRA), which provided cervical cancer screening services for eligible women in rural areas for free 17. The program screened about 10 million women aged 35–59 years from 2009 to 2011; since then, the program screened about 10 million rural women aged 35–64 years every year 18, 19. Nevertheless, the program could only serve a small fraction of eligible women and most of the eligible women could only seek services by themselves (approximately 245 million women aged 35–64 years in China) 18. As a result, an alarmingly high percentage of women reported never having had a Pap smear previously 20, indicating the necessity of identifying priority groups for an effective delivery strategy 21.

In this study, our objective was to investigate the significant variations in cervical cancer screening rates in China based on SES and geographical regions by utilizing a nationally representative, cross‐sectional survey dataset between August 2013 and July 2014. Of particular interest was whether cervical cancer screening is associated with both individual‐level and geographical measures of SES concurrently; such associations would help to identify priority populations for target interventions.

## Methods

### Study design

This was a cross‐sectional study conducted in China between August 2013 and July 2014 as part of the Chinese Chronic Disease and Risk Factor Surveillance (CCDRFS), which is a nationally representative, multipurpose survey conducted every 3 years 22. Of total 98,756 women interviewed in this survey, 97,942 subjects aged 21 years or older were included in this analysis according to recommendations from the American Congress of Obstetricians and Gynecologists 23. The surveillance system adopted a multistage stratified cluster sampling strategy and randomly selected 297 districts or counties as primary sampling units (PSU) from 31 provinces, autonomous regions, and municipalities in mainland China, with stratification by population size and mortality rate of each province. Within PSU, a four‐stage random cluster sampling method was applied for the selection of individuals: stage I, four townships were selected by the method of probability‐proportional‐to‐size sampling in each PSU; stage II, three communities or villages were selected in each chosen township using the same sampling method employed in stage I; stage III, a residential group composed of at least 50 households was selected in each of the communities or villages using simple random sampling; stage IV, one eligible member in each family was selected as a research subject using a Kish selection table. The eligible members included those aged 18+ years who resided in the survey areas at least for 6 months in the 12 months prior to the survey. Face‐to‐face interviews were conducted by trained investigators using a unified questionnaire. If selected families or members were not accessed or refused the survey, they were replaced by others having a similar family structure. Approximately 6% of the sampled families were replaced.

### Measures

The interview included questions about both demographic and socioeconomic characteristics, and the uptake of cervical cancer screening services. Respondents were asked whether they had ever had at least one cervical cancer screening; where applicable, women were asked when they had received their last examination. Four individual‐level variables, education attainment, types of employment, household wealth, and types of medical insurance, were used as indicators of individual‐level measures of SES 24. Educational attainment was divided into three categories: lowest, individuals with primary school education and lower; medium, individuals with secondary school education (junior/senior high school); and highest, individuals with at least some postsecondary education (university/college/postgraduate). The types of employment were categorized as unemployed (housewife/student/unemployed), agriculture employment, nonagriculture employment, and retired. The household wealth was represented by annual household income and categorized into quartiles (low‐2687, 2687–4478, 4478–7463, and 7463‐high U.S. dollars) and those with an unknown household income (refused/don't know). Medical insurance was divided into five categories: medical insurance for urban employment; medical insurance for urban unemployment; new rural cooperative medical scheme (NCMS) for the rural residents; others (e.g., commercial insurance); and no insurance.

Three geographical variables, serving as proxies for geographical measure of SES 25, 26, were collected from the 2010 Chinese population census and linked with residential address of each woman interviewed. They were (1) county‐level urbanization represented by the percentage of residents living in the urban areas, (2) county‐level education status represented by the percentage of individuals aged ≥25 years who are college graduates, (3) province‐level economic status represented by per capita gross domestic product (GDP). Each variable was broken into quintiles.

### Statistical analyses

Cervical cancer screening rate was defined as the percentage of eligible women who reported ever having screened for cervical cancer at least once before the survey. Weighted rates with 95% confidence intervals (CI) were estimated for all participants and for women aged 35–64 years, in 31 provincial administrative units in mainland China (including 22 provinces, four municipalities, and five autonomous regions, but not including Hong Kong, Macao Special Administrative Regions, and Taiwan), and in subgroups of individual‐level and geographical SES variables, taking account of complex sampling design. Weights included sampling weight and poststratified weight from the 2010 Chinese population census to adjust for differences in probability of selection, nonresponse, and noncoverage. The rates of geographical regions were mapped into a visual geographical map. Single‐level, age‐adjusted logistic regression models were fitted for trend analysis of each individual‐level or geographical SES variable. All these estimations were obtained using SAS 9.4 (SAS Institute Inc., Cary, USA).

To investigate the geographical variations and the association of cervical cancer screening with both individual‐level and geographical SES, a series of multilevel logistic regression models with random intercepts were fitted according to three levels: individual (level 1, *n* = 91,816), county (level 2, *n* = 297), and provincial administrative units (level 3, *n* = 31). The outcome measure was the uptake of cervical cancer screening, and the explanatory variables included covariates (age, marital status, ethnicity, and place of residence), individual‐level measures of SES, and geographical measures of SES. A null model without independent variables was carried out to compute the geographical variations, and then, individual‐level variables, geographical variables, and interaction terms were successively added in models. The fixed effects of all explanatory variables were converted into odd ratios (OR) with 95% CIs. Random intercepts, represented by variance and standard error (SE) at each level, accounted for geographical variations between counties (level 2) and between provinces (level 3). A *P* < 0.05 of random effect indicated a significant variation in cervical cancer screening between geographical regions. Percentage of change in variance (PCV), or the percentage of variance explained by independent variables, was also calculated 27. All parameters were tested using Wald tests 28, and statistical significance was defined as a *P*‐value less than 0.05 using a two‐sided test. These models were conducted by using MLwiN version 2.30.

## Results

### Characteristics of eligible women

Of the 97,942 interviewed women aged at least 21 years, 91,816 (response rate = 93.7%) were eligible for the study (Table 1). The characteristics of participants included and excluded in the analyses are presented in Table S1. The mean age of eligible women was 51.7 (standard deviation = 13.5) years, and 52.1% lived in rural areas, 86.4% were married, 54.1% had a primary school education or lower, 26.7% were unemployed, and 2.3% were uninsured.

**Table 1:** Demographic and socioeconomic characteristics of eligible participants aged 21 years or older, China, 2013–2014

Characteristic	No. of eligible participants	Unweighted proportion % (95% CI)	Weighted proportion % (95% CI)
Total	91,816	100.0	100.0
Age (years)			
21–29	5991	6.5 (6.0–7.0)	22.2 (20.5–23.8)
30–39	12,138	13.2 (12.7–13.8)	21.3 (20.5–22.0)
40–49	24,472	26.7 (26.0–27.3)	23.0 (22.3–23.8)
50–59	24,315	26.5 (25.9–27.0)	16.0 (15.3–16.7)
60–69	16,937	18.4 (17.8–19.0)	9.7 (9.2–10.3)
More than 70	7963	8.7 (8.2–9.2)	7.8 (7.0–8.6)
Residence			
Urban	43,980	47.9 (45.0–50.8)	46.7 (42.7–50.6)
Rural	47,836	52.1 (49.2–55.0)	53.3 (49.4–57.3)
Race			
Han	81,873	89.2 (87.5–90.9)	91.4 (88.8–94.0)
Others	9905	10.8 (9.1–12.5)	8.6 (6.0–11.2)
Marital status			
Married	79,234	86.4 (85.7–87.0)	86.6 (85.6–87.6)
Never married	1879	2.0 (1.8–2.3)	6.3 (5.4–7.2)
Other	10,644	11.6 (11.0–12.2)	7.1 (6.6–7.8)
Education			
Primary school and lower	49,617	54.1 (52.1–56.0)	44.2 (41.7–46.6)
Secondary school	36,916	40.2 (38.7–41.7)	46.1 (44.3–47.8)
Some postsecondary	5236	5.7 (4.9–6.5)	9.7 (8.1–11.5)
Household wealth			
Refused/do not know	21,843	23.8 (22.1–25.6)	23.7 (21.0–26.2)
Q1	18,734	20.4 (19.0–21.9)	16.1 (14.3–17.9)
Q2	18,465	20.1 (19.3–21.0)	19.5 (18.2–20.9)
Q3	16,482	18.1 (17.1–18.8)	19.2 (17.9–20.6)
Q4	16,116	17.6 (16.3–18.9)	21.5 (19.4–23.6)
Type of employment			
Unemployed	24,511	26.7 (24.8–28.7)	26.5 (24.5–28.5)
Employed	59,100	64.3 (62.4–66.4)	68.3 (66.2–70.4)
Retired	8158	9.0 (7.4–10.4)	5.2 (4.0–6.5)
Types of medical insurance			
Insurance for urban employment	16,766	18.3 (16.0–20.5)	18.0 (15.0–21.0)
Insurance for urban unemployment	9246	10.2 (8.9–11.2)	8.5 (7.3–9.8)
NCMS	63,098	68.7 (65.7–71.8)	69.8 (65.8–73.8)
Others	478	0.5 (0.4–0.6)	0.6 (0.4–0.7)
No insurance	2148	2.3 (2.1–2.6)	3.1 (2.7–3.6)

### Variations in cervical cancer screening rates

Overall, an estimated 21.4% (95% CI: 19.6–23.1%) of women aged at least 21 years reported having undergone cervical cancer screening previously and the screening rate was 26.7% (95% CI: 24.6–28.9%) among women aged 35–64 years (Table 2). Women between the ages of 30 and 49 years were more likely to have undergone screening than those in the other groups. The screening rate among women residing in urban areas (25.2%, 95% CI: 23.0–27.4%) was substantially higher than those residing in rural areas (18.0%, 95% CI: 16.2–19.8%). The highest screening rate was observed among women aged 40–49 years in urban areas (35.7%, 95% CI: 32.8–38.6%).

**Table 2:** The cervical cancer screening rates among women with aged 21 years and older, by age, and residence, China, 2013–2014

Age of respondent (years)	All		Urban		Rural	
	No. of ever had screening/no. of sample^a^	% (95% CI)^b^	No. of ever had screening/no. of sample	% (95% CI)	No. of ever had screening/no. of sample	% (95% CI)
21–29	883/5991	12.1 (10.3–13.9)	411/2862	12.5 (10.0–14.9)	472/3129	11.7 (9.3–14.2)
30–39	3533/12,138	29.6 (27.0–32.2)	1907/5754	35.1 (31.4–38.8)	1626/6384	24.5 (22.0–26.9)
40–49	7616/24,472	30.8 (28.4–33.2)	3914/10,878	35.7 (32.8–38.6)	3702/13,594	26.8 (24.1–29.4)
50–59	5990/24,315	22.7 (20.4–25.0)	3477/11,885	27.9 (25.1–30.8)	2513/12,430	18.1 (15.6–20.6)
60–69	2314/16,937	12.3 (10.4–14.3)	1489/8445	16.5 (14.0–19.0)	825/8492	8.8 (6.6–11.0)
70–high	527/7963	6.0 (4.6–7.5)	393/4156	9.2 (6.6–11.9)	134/3807	3.4 (2.6–4.8)
35–64	17,376/66,130	26.7 (24.6–28.9)	9570/31,174	31.8 (29.2–34.3)	7806/34,956	22.4 (20.1–24.8)
Total	20,863/91,816	21.4 (19.6–23.1)	11,591/43,980	25.2 (23.0–27.4)	9272/47,836	18.0 (16.2–19.8)

CI, Confidence interval.

a Number refers to unweighted sample.

b Weighted rates.

**Figure 1:**
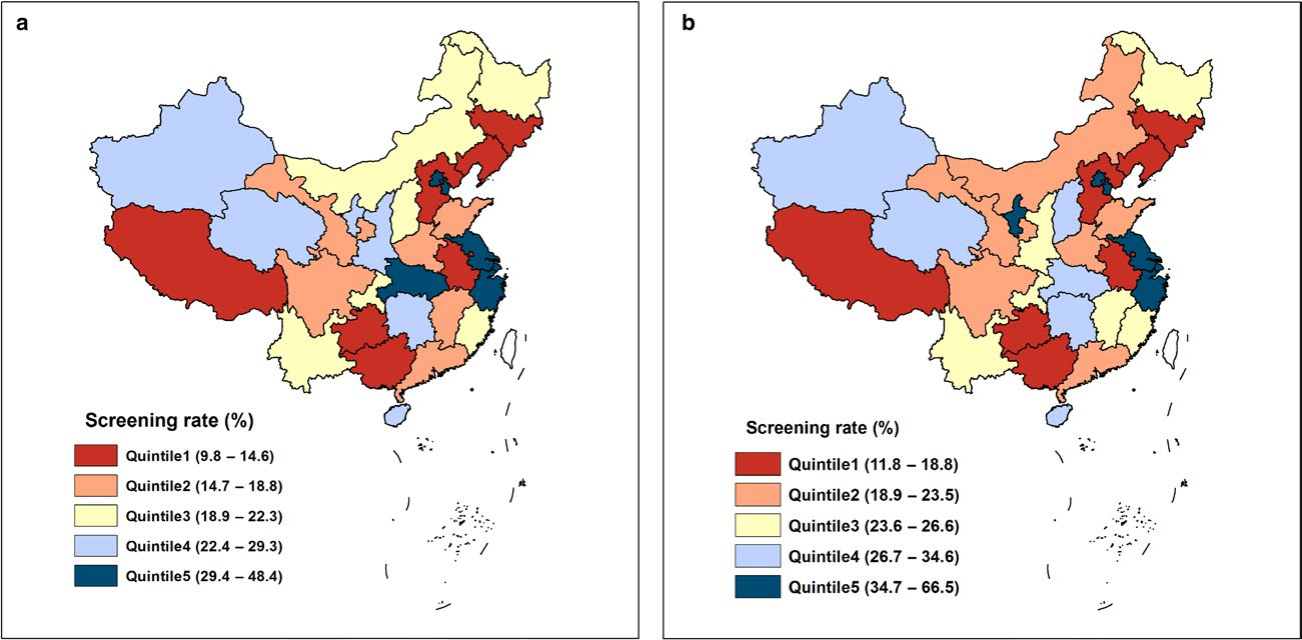
Cervical cancer screening rates by 31 provincial administrative units in mainland China in 2013–2014 [(a) screening rates among women aged at least 21 years; (b) screening rates among women aged 35–64 years). All rates were weighted by provincial population. The 31 provincial administrative units (not including Hong Kong, Macao Special Administrative Regions, and Taiwan) were broken into quintiles according to the screening rates.

As shown in Figure 1, the cervical cancer screening rates among women aged at least 21 years ranged from 48.4% in Beijing to 9.8% in Tibet (Fig. 1a & Table S2). Five (Beijing, Zhejiang, Shanghai, Tianjin, and Jiangsu) of 31 provincial administrative units had achieved cervical cancer screening coverage above 30%. The provinces with coverage below 20% were clustered in western and central China. Among women aged 35–64 years, the cervical cancer screening rates ranged from 66.5% in Beijing to 11.8% in Tibet and approximately one‐third of the provinces reported above 30% coverage (Fig. 1b & Table S2).

Table 3 shows the positive gradients of individual‐level and geographical measure of SES for cervical cancer screening rates. At an individual level, cervical cancer screening rates were the lowest among the poor (13.9%), uninsured (14.4%), less‐educated (16.0%), and agricultural employment (18.1%) group. At a geographical level, the lowest rates of cervical cancer screening were observed in areas of low economic (15.0%) status, low urbanization (15.6%), and low education (16.0%).

**Table 3:** Variations in cervical cancer screening rates by individual‐level and geographical measures of SES

Socioeconomic status	No. of ever had screening/no. of sample^a^	%(95% CI)^b^	OR (95% CI) Ever v never cervical cancer screening
Individual level			
Education attainment			
Primary school and lower	8010/49,617	16.0 (14.3–17.6)	0.56 (0.45–0.69)
Secondary school	10,906/36,916	25.4 (23.3–27.6)	0.94 (0.79–1.12)
Some postsecondary	1934/5236	26.6 (23.1–30.0)	1.00 (reference)
*P*_*trend*_ value			<0.0001
Type of employment			
Nonagriculture employment	6206/18,705	28.7 (25.9–31.5)	1.00 (reference)
Agricultural employment	7607/40,395	18.1 (15.8–20.4)	0.65 (0.53–0.79)
Retired	2238/8158	25.8 (21.3–30.4)	0.91 (0.72–1.14)
Unemployed	4801/24,511	18.2 (16.6–19.7)	0.59 (0.52–0.67)
*P*_*trend*_ value			<0.0001
Type of medical insurance			
Insurance for urban employment	5653/16,766	32.9 (29.3–36.4)	1.00 (reference)
Insurance for urban unemployment	2339/9246	24.5 (22.0–26.9)	0.65 (0.56–0.76)
NCMS	12,341/63,098	18.3 (16.5–20.1)	0.49 (0.40–0.60)
Other	132/478	26.7 (18.3–35.2)	0.77 (0.51–1.15)
No insurance	309/2148	14.4 (10.3–18.4)	0.34 (0.25–0.47)
*P*_*trend*_ value			<0.0001
Household wealth			
Refused/do not know	3986/21,843	19.1 (16.8–21.4)	0.63 (0.53–0.74)
Q1 (lowest)	3138/18,734	13.9 (12.1–15.7)	0.47 (0.39–0.58)
Q2	4259/18,465	20.8 (19.0–22.7)	0.73 (0.64–0.83)
Q3	4303/16,482	22.8 (21.0–24.6)	0.78 (0.70–0.87)
Q4 (highest)	5138/16,116	28.7 (25.5–31.9)	1.00 (reference)
*P*_*trend*_ value			<0.0001
Geographical level			
Urbanization			
Q1 (lowest)	2263/16,430	15.6 (13.4–17.7)	0.47 (0.35–0.62)
Q2	3741/18,591	16.7 (14.2–19.3)	0.51 (0.38–0.69)
Q3	4412/18,471	23.8 (18.1–29.5)	0.79 (0.55–1.13)
Q4	4482/18,826	23.4 (19.8–26.9)	0.77 (0.56–1.05)
Q5 (highest)	5565/19,498	28.4 (25.7–31.0)	1.00 (reference)
*P*_*trend*_ value			<0.0001
Education status			
Q1 (lowest)	2466/17,963	16.0 (14.0–18.1)	0.46 (0.34–0.61)
Q2	3433/17,950	19.7 (17.7–21.7)	0.58 (0.40–0.84)
Q3	4149/17,870	18.2 (15.6–20.9)	0.53 (0.40–0.71)
Q4	4991/18,306	25.1 (21.8–28.3)	0.79 (0.58–1.06)
Q5 (highest)	5824/19,727	29.6 (26.8–32.4)	1.00 (reference)
*P*_*trend*_ value			<0.0001
Economic status			
Q1 (lowest)	2509/15,875	15.0 (11.8–18.2)	0.42 (0.29–0.60)
Q2	3775/18,170	18.3 (15.2–21.4)	0.50 (0.35–0.70)
Q3	4286/18,849	22.2 (19.4–25.0)	0.62 (0.46–0.85)
Q4	3869/19,943	18.6 (16.0–21.2)	0.49 (0.36–0.68)
Q5 (highest)	6424/18,979	31.7 (25.4–37.9)	1.00 (reference)
*P*_*trend*_ value			<0.0001

OR, Odds ratio; CI, confidence interval. It is estimated by one‐level, only age‐adjusting multivariate logistic regression.

a Number refers to unweighted sample.

b Weighted rates.

### Association of cervical cancer screening with both individual‐level and geographical measures of socioeconomic status

Results of multilevel analyses from four models are summarized in Table 4. Significant geographical variations were identified between provinces (*P* = 0.0011) and between countries (*P* < 0.0001) (Model 1). The likelihood of utilization of cervical cancer screening was lower among women who had primary education (*P* < 0.0001), no insurance (*P* < 0.0001), were unemployed (*P* < 0.0001), low household wealth (*P* < 0.0001), and those residing in areas with the lowest education (*P* = 0.0049) and economic (*P* = 0.0006) status (Model 3). There were significant positive interactions between geographical education status and individual education level and between geographical economic status and individual employment (Model 4). In total, approximately 12% and 49% of county‐level and province‐level variations were explained, respectively; however, county‐level and province‐level intercept variances remained strongly significant, showing that marked geographical variations still existed between counties and between provinces after controlling for individual‐level and geographical measures of SES.

**Table 4:** Multilevel analyses for the associations of cervical cancer screening with both individual‐level and geographical measures of SES^a^ ^1^

Variables	Model 1		Model 2		Model 3		Model 4	
	OR (95% CI)	*P* value	OR (95% CI)	*P* value	OR (95% CI)	*P* value	OR (95% CI)	*P* value
Fixed effects								
Individual‐level SES								
Age group								
21–29 years			1.00 (reference)		1.00 (reference)		1.00 (reference)	
30–39 years			2.21 (2.02–2.43)	<0.0001	2.21 (2.02–2.43)	<0.0001	2.21 (2.01–2.42)	<0.0001
40–49 years			2.69 (2.46–2.95)	<0.0001	2.69 (2.45–2.94)	<0.0001	2.67 (2.44–2.92)	<0.0001
50–59 years			1.79 (1.64–1.97)	<0.0001	1.79 (1.63–1.96)	<0.0001	1.77 (1.62–1.94)	<0.0001
60–69 years			0.88 (0.80–0.98)	0.0159	0.88 (0.79–0.97)	0.0137	0.87 (0.79–0.96)	0.0082
70‐plus			0.39 (0.34–0.44)	<0.0001	0.39 (0.34–0.44)	<0.0001	0.39 (0.34–0.44)	<0.0001
Marital status								
Married			1.00 (reference)		1.00 (reference)		1.00 (reference)	
Never married			0.20 (0.16–0.24)	<0.0001	0.20 (0.16–0.24)	<0.0001	0.20 (0.17–0.24)	<0.0001
Other			0.83 (0.77–0.89)	<0.0001	0.83 (0.77–0.89)	<0.0001	0.83 (0.78–0.89)	<0.0001
Residence								
Urban			1.00 (reference)		1.00 (reference)		1.00 (reference)	
Rural			0.98 (0.94–1.03)	0.5194	0.99 (0.94–1.04)	0.6122	0.99 (0.94–1.04)	0.6213
Ethnicity								
Han			1.00 (reference)		1.00 (reference)		1.00 (reference)	
Other			0.89 (0.82–0.97)	0.0047	0.90 (0.83–0.97)	0.0072	0.90 (0.83–0.97)	0.0064
Education attainment								
Primary school and lower			0.47 (0.43–0.52)	<0.0001	0.47 (0.43–0.52)	<0.0001	0.45 (0.40–0.52)	<0.0001
Secondary school			0.74 (0.68–0.80)	<0.0001	0.74 (0.69–0.80)	<0.0001	0.76 (0.69–0.84)	<0.0001
Some postsecondary			1.00 (reference)		1.00 (reference)		1.00 (reference)	
Type of medical insurance								
Insurance for urban employment			1.00 (reference)		1.00 (reference)		1.00 (reference)	
Insurance for urban unemployment			0.79 (0.73–0.84)	<0.0001	0.79 (0.73–0.84)	<0.0001	0.78 (0.73–0.84)	<0.0001
NCMS			0.76 (0.70–0.81)	<0.0001	0.76 (0.71–0.82)	<0.0001	0.76 (0.70–0.82)	<0.0001
Other			0.73 (0.58–0.92)	0.0075	0.73 (0.58–0.92)	0.0076	0.73 (0.58–0.92)	0.0074
No insurance			0.47 (0.41–0.54)	<0.0001	0.47 (0.41–0.54)	<0.0001	0.46 (0.40–0.53)	<0.0001
Type of employment								
Nonagriculture employment			1.00 (reference)		1.00 (reference)		1.00 (reference)	
Agricultural employment			0.86 (0.81–0.91)	<0.0001	0.86 (0.81–0.92)	<0.0001	0.97 (0.86–1.09)	0.6108
Retired			0.96 (0.89–1.03)	0.2451	0.95 (0.88–1.03)	0.2235	1.07 (0.95–1.21)	0.2659
Unemployed			0.78 (0.74–0.83)	<0.0001	0.78 (0.74–0.83)	<0.0001	0.92 (0.82–1.02)	0.1111
Household wealth								
Refused/don't know			0.77 (0.72–0.81)	<0.0001	0.77 (0.72–0.82)	<0.0001	0.77 (0.72–0.81)	<0.0001
Q1			0.84 (0.79–0.90)	<0.0001	0.85 (0.79–0.90)	<0.0001	0.84 (0.79–0.90)	<0.0001
Q2			0.97 (0.91–1.03)	0.2508	0.97 (0.91–1.03)	0.2865	0.97 (0.91–1.02)	0.2341
Q3			0.96 (0.91–1.01)	0.1220	0.96 (0.91–1.01)	0.1298	0.96 (0.90–1.01)	0.1062
Q4			1.00 (reference)		1.00 (reference)		1.00 (reference)	
Geographical SES								
County‐level urbanization								
Q1 (lowest)					1.03 (0.62–1.70)	0.9092	1.03 (0.63–1.70)	0.8993
Q2					1.41 (0.88–2.27)	0.1582	1.41 (0.88–2.26)	0.1587
Q3					1.35 (0.87–2.11)	0.1868	1.36 (0.87–2.12)	0.1750
Q4					1.09 (0.76–1.57)	0.6249	1.10 (0.77–1.58)	0.5889
Q5 (highest)					1.00 (reference)		1.00 (reference)	
County‐level education status								
Q1 (lowest)					0.49 (0.30–0.80)	0.0049	0.89 (0.49–1.58)	0.6801
Q2					0.58 (0.36–0.92)	0.0207	0.55 (0.31–0.96)	0.0341
Q3					0.68 (0.43–1.07)	0.0985	0.74 (0.44–1.25)	0.2562
Q4					0.93 (0.65–1.34)	0.6976	0.82 (0.56–1.21)	0.3171
Q5 (highest)					1.00 (reference)		1.00 (reference)	
Province‐level economic status								
Q1 (lowest)					0.41 (0.25–0.68)	0.0006	0.56 (0.33–0.96)	0.0329
Q2					0.65 (0.39–1.08)	0.0944	0.76 (0.45–1.27)	0.2914
Q3					0.68 (0.42–1.12)	0.1316	0.70 (0.42–1.17)	0.1766
Q4					0.51 (0.31–0.84)	0.0074	0.56 (0.34–0.93)	0.0245
Q5 (highest)					1.00 (reference)		1.00 (reference)	
Interaction between individual‐level and geographical SES								
County‐level education status and individual education								
Q5 (highest) × postsecondary							1.00 (reference)	
Q1 (lowest) × primary school and lower							0.55 (0.39–0.76)	0.0004
Q1 (lowest) × secondary school							0.55 (0.40–0.76)	0.0003
Province‐level economic status and individual type of employment								
Q5 (highest) × nonagriculture employment							1.00 (reference)	
Q1 (lowest) × agricultural employment							0.67 (0.56–0.81)	<0.0001
Q1 (lowest) × Unemployed							0.63 (0.52–0.77)	<0.0001
Random effects								
Variance between counties (SE)	0.620 (0.056)	<0.0001	0.597 (0.055)	<0.0001	0.552 (0.051)	<0.0001	0.547 (0.050)	<0.0001
PCV (%)			3.7	–	11.0	–	11.8	–
Variance between provinces (SE)	0.286 (0.091)	0.0011	0.298 (0.094)	0.0014	0.145 (0.053)	0.0069	0.146 (0.054)	0.0063
PCV (%)			−4.1	–	49.3	–	49.0	–

OR, odds ratio; CI, confidence interval; SE, standard error; PCV, percentage of change in variance.

a All women aged 21+ years are included in models and 369 women with missing values on socioeconomic variables omitted from analysis.

## Discussion

Based on a large, nationally representative survey in 2013 and 2014, there was an alarmingly low cervical cancer screening rate and marked variations for individual‐level and geographical measures of SES. The updated screening rate for cervical cancer among Chinese women aged 21 years or older in our latest survey was similar to that from the previous survey in 2010, that is, 21.4% versus 20.7% 20. With approximately 500 million women aged at least 21 in mainland China in 2010, it is projected that 394 million women were never screened for cervical cancer before the survey. Unscreened women were most common in populations with lower individual‐level measures of SES who were residing in areas with lower geographical measures of SES. Compared with that in other cross‐sectional studies, the screening rate in China is markedly lower than those in developed countries, such as the UK (78%) 29, Finland (79.2%) 30, and Spain (65.6%) 31; additionally, it is lower than those in neighboring countries, such as Korea (75.5%) 32, Thailand (67.4%) 33, and Japan (32.0%) 34.

The rates of cervical cancer screening varied substantially across geographical areas, with screening rates ranging from <10% to >40% across the 31 Chinese provincial administrative units. The geographical patterns were similar between women aged ≥21 years and women aged 35–64 years, suggesting no transition of wide variations within age subgroups. Differences in socioeconomic development, local health policy, and cultural background in China may be attributed to these disparities across regions 35. Further, these findings are completely opposite to the geographical patterns of cervical cancer mortality in that the death rates of cervical cancer in central and western areas are higher than those in coastal areas 36, 37. The large gap between geographical patterns of screening rates and death rates should be considered in the implementation of government‐funded cervical cancer screening programs accordingly.

Our study demonstrated that less‐educated, unemployed, poor, or uninsured women were less likely to undergo screening for cervical cancer, which is consistent with the findings from previous studies 38, 39. The association of education attainment with cervical cancer screening could be attributed to the linkage between education level and access to information about cancer screening or the capacity to make appropriate decisions 6, 40. The occupational class and household wealth are related to the affordability of cervical cancer screening. Many studies found that financial barriers to screening remained among most deprived women even in some developed countries 41, 42. As for medical insurance, higher reimbursement rates may increase clinic visits and thus increase the likelihood of opportunistic screening test 9, 43. To address these barriers, interventions should be targeted toward women never screened and outreach may be an effective strategy for organized screening for such women as they are at the highest risk of developing invasive cervical cancer 21, 40.

An important finding from the current study is that geographical measures of SES, that is, education and economic status, may be positively associated with cervical cancer screening, as also found by Nathalie et al. 11. Furthermore, the finding that individual‐level measures of SES may be modified by area‐based measures of SES by Coughlin et al. was also observed in our study 14. The significant interaction between individual‐level and geographical variables in our study suggests the priority populations for organized cervical cancer screening in future. Specifically, women with low education levels, residing in areas with a higher percentage of low‐educated women, and low‐class occupation women residing in areas of low economic levels were significantly less likely to undergo cancer screening, compared with corresponding women residing in other advantaged areas. The contextual effect may be due to the socioeconomic factors in addition to a lack of culturally appropriate and accessible preventive healthcare service in these areas 42, for example, notable advocacy and government‐funded programs for cancer prevention in high‐income areas, but the neglect of gynecological cancers in low‐income areas 44. Healthcare workers may play an important role in the cervical cancer screening examination, and their numbers and competency levels are significantly determined by regional socioeconomic status 13. It is estimated that there are 10.2 healthcare workers per 1000 in urban China, but only 3.9 per 1000 in rural areas and a large gap remains between competence of the healthcare workers in urban and rural settings 45. However, reasons for the association of geographical variables with cervical cancer screening are difficult to determine from cross‐sectional surveillance data and further investigation including additional variables about detailed area‐based characteristics are required to determine the causes. Regardless, our findings still provide guidance as to the potential needs of directing finite organized cervical cancer screening services to priority populations in specific areas in low‐resource settings.

To our knowledge, this is the first study to investigate the variations in cervical cancer screening rates in China, using both individual‐level and geographical measures of SES as predictors. Our study benefits from a large nationally representative sample of the general population in China, following a strict sampling design and quality control surveillance to ensure data validity and reliability. Without population‐based record systems for cervical cancer screening, the current data are the sole access to the estimation of population‐based screening rate at a national scale. Random intercept with variance and error is also used to quantitatively calculate the geographical variations in cervical cancer screening rates.

The current study had several limitations. The study is unavoidably open to response bias because about 6% of sampled women without cervical cancer screening data were excluded. The demographic and socioeconomic characteristics of the included and excluded subjects are substantially different and could affect the estimates. Moreover, self‐reported information about cancer screening instead of records from clinical practice may involve recall bias. A further issue is that we were not able to confirm whether subjects receive the screening service through the organized program or a daily clinical visit.

## Conclusions

In this study, we investigate the significant variations in the cervical cancer screening rates based on geographical regions and multilevel measures of SES using a cross‐sectional, nationally representative survey dataset. Public health resources are so limited that the government‐funded NACCSPRA serves only a small fraction of eligible women in China, and our study focuses on women who had never been screened. We found that socioeconomically disadvantaged women residing in areas of socioeconomic deprivation were less likely to undergo cervical cancer screening. These findings suggest that it should be a priority to better direct finite screening resources to this group of women.

## Conflicts of Interest

The authors declare that they have no competing interests.

## Ethical Consideration

The ethics committee of the Chinese Centers for Disease Control and Prevention approved the study and all participants were well informed about the study and provided written consent.

## Supporting information


**Table S1.** Basic socioeconomic characteristics of eligible and refusal women in survey.
**Table S2.** The cervical cancer screening rates and 95% CI among women by provincial administrative units, China, 2013–2014.
