# Supplementary material for: Significant variations in the cervical cancer screening rate in China by individual‐level and geographical measures of socioeconomic status: a multilevel model analysis of a nationally representative survey dataset
Source: Cancer Med. 2018 Mar 24;7(5):2089–100. doi: 10.1002/cam4.1321 (PMC5943548; doi:10.1002/cam4.1321)
Supplement: Supplementary file 1 — Table S1. Basic socioeconomic characteristics of eligible and refusal women in survey. Table S2. The cervical cancer screening rates and 95% CI among women by provincial administrative units, China, 2013–2014. [file CAM4-7-2089-s001.docx]

**Table S1.** Basic socioeconomic characteristics of eligible and refusal women in survey.

| **Characteristic** | **No. of eligible for cervical cancer screening (unweighted proportion %)** | **No. of refusals or contact failure**  **(unweighted proportion %)** | ***P*-value** |
| --- | --- | --- | --- |
| **Total** | 91816 (100.0) | 6126 (100.0) |  |
| **Age (years)** |  |  | <0.0001 |
| 21-29 | 5991 (6.5) | 350 (5.6) |  |
| 30-39 | 12138 (13.2) | 647 (10.6) |  |
| 40-49 | 24472 (26.7) | 1230 (20.1) |  |
| 50-59 | 24315 (26.5) | 1390 (22.7) |  |
| 60-69 | 16937 (18.4) | 1408 (23.0) |  |
| More than 70 | 7963 (8.7) | 1101 (18.0) |  |
| **Residence** |  |  | 0.9449 |
| urban | 43980 (47.9) | 2686 (43.8) |  |
| rural | 47836 (52.1) | 3440 (56.2) |  |
| **Race** |  |  | 0.2811 |
| Han | 81873 (89.2) | 5092 (83.2) |  |
| Others | 9905 (10.8) | 1031 (16.8) |  |
| **Marital status** |  |  | 0.0003 |
| Married | 79234 (86.4) | 5037 (82.2) |  |
| Never married | 1879 (2.0) | 118 (2.0) |  |
| Other | 10644 (11.6) | 966 (15.8) |  |
| **Education attainment** |  |  | <0.0001 |
| Primary school and lower | 49617 (54.1) | 4154 (67.8) |  |
| Secondary school | 36916 (40.2) | 1736 (28.4) |  |
| Some postsecondary | 5236 (5.7) | 233 (3.8) |  |
| **Household wealth** |  |  | <0.0001 |
| Don’t know/refused | 21843 (23.8) | 2271 (37.1) |  |
| Q1 (low-18000) | 18734 (20.5) | 1240 (20.3) |  |
| Q2 (18000-30000) | 18465 (20.1) | 884 (14.5) |  |
| Q3 (30000-50000) | 16482 (18.1) | 808 (13.2) |  |
| Q4 (50000-high) | 16116 (17.6) | 911 (14.9) |  |
| **Type of employment** |  |  | 0.1314 |
| Unemployed | 24511 (26.7) | 1672 (27.3) |  |
| Employed | 59100 (64.3) | 3849 (62.9) |  |
| Retired | 8158 (9.0) | 603 (9.8) |  |
| **Type of medical insurance** |  |  | 0.2945 |
| Insurance for urban employment | 16766 (18.3) | 931 (15.2) |  |
| Insurance for urban unemployment | 9246 (10.2) | 654 (10.7) |  |
| NCMS | 63098 (68.7) | 4340 (70.9) |  |
| Others | 478 (0.5) | 41 (0.7) |  |
| No insurance | 2148 (2.3) | 156 (2.5) |  |

**Table S2.** The cervical cancer screening rates and 95%CI among women by provincial administrative units, China, 2013-2014.

| **Province** | **Women aged 21 years and over** | |  | **Women aged 35-64 years** | |
| --- | --- | --- | --- | --- | --- |
|  | **No. of ever had screening  /No. of Sample** | **Weighted  % (95%CI)** |  | **No. of ever had screening  /No. of Sample** | **Weighted  % (95%CI)** |
| Beijing | 1293/2349 | 48.4 (39.4-57.5) |  | 1143/1706 | 66.5 (56.6-76.4) |
| Zhejiang | 1297/2944 | 43.2 (26.3-60.1) |  | 1045/2059 | 51.9 (33.9-70.0) |
| Shanghai | 646/1905 | 35.3 (23.8-46.8) |  | 519/1219 | 47.1 (33.4-60.8) |
| Tianjin | 853/2082 | 34.4 (18.7-50.0) |  | 753/1515 | 52.3 (33.3-71.2) |
| Jiangsu | 1399/4103 | 32.7 (15.3-50.2) |  | 1092/2853 | 39.1 (17.1-61.1) |
| Hubei | 870/3252 | 29.6 (20.3-38.8) |  | 696/2354 | 34.7 (24.4-45.0) |
| Qinghai | 523/1939 | 29.4 (13.1-45.7) |  | 432/1517 | 31.2 (13.1-49.2) |
| Ningxia | 607/1864 | 28.9 (14.0-42.8) |  | 510/1380 | 37.9 (22.8-53.1) |
| Hainan | 499/1269 | 25.6 (21.8-29.3) |  | 418/1271 | 34.7 (30.2-39.3) |
| Hunan | 1060/4078 | 25.1 (19.1-31.2) |  | 914/2938 | 34.5 (26.7-42.2) |
| Xinjiang | 570/2195 | 23.7 (14.0-33.5) |  | 467/1480 | 31.5 (19.9-43.2) |
| Shaanxi | 686/2975 | 22.6 (10.9-34.3) |  | 555/2090 | 26.3 (10.5-42.1) |
| Yunnan | 726/3065 | 22.4 (16.4-28.3) |  | 585/2287 | 26.2 (19.6-32.8) |
| Shanxi | 614/2575 | 21.9 (13.8-30.0) |  | 529/1971 | 28.5 (17.4-39.7) |
| Fujian | 737/3138 | 21.7 (16.2-27.2) |  | 639/2403 | 26.7 (14.7-38.7) |
| Chongqing | 563/3134 | 19.7 (14.9-24.5) |  | 477/2161 | 24.5 (16.0-33.0) |
| Inner Mongolia | 524/2531 | 19.3 (5.2-33.4) |  | 440/1960 | 21.8 (4.7-38.9) |
| Heilongjiang | 518/3178 | 19.0 (16.4-21.5) |  | 442/2325 | 24.2 (19.8-28.5) |
| Sichuan | 620/3895 | 18.9 (12.1-25.7) |  | 499/2612 | 22.1 (15.0-29.2) |
| Guangdong | 761/4297 | 18.8 (12.5-25.0) |  | 642/3111 | 23.6 (15.4-31.9) |
| Jiangxi | 747/3291 | 16.8 (13.3-20.3) |  | 638/2439 | 25.0 (17.7-32.2) |
| Shandong | 717/3887 | 16.8 (11.9-21.7) |  | 576/2703 | 22.1 (14.5-29.7) |
| Henan | 772/4702 | 16.1 (8.8-23.3) |  | 637/3379 | 20.5 (10.1-31.0) |
| Gansu | 501/2598 | 15.9 (10.1-21.7) |  | 439/1988 | 19.1 (13.0-25.2) |
| Hebei | 661/4282 | 14.7 (12.1-17.4) |  | 543/3051 | 18.0 (14.3-21.8) |
| Jilin | 405/2512 | 14.3 (2.4-26.2) |  | 360/1874 | 18.9 (4.1-33.7) |
| Liaoning | 412/3065 | 13.6 (8.8-18.4) |  | 351/2009 | 16.7 (9.8-23.6) |
| Anhui | 414/3678 | 13.1 (2.7-23.6) |  | 327/2557 | 14.5 (3.6-25.5) |
| Guizhou | 236/2226 | 12.7 (6.6-18.7) |  | 195/1560 | 16.0 (8.6-23.5) |
| Guangxi | 425/3151 | 11.6 (7.8-15.5) |  | 348/2143 | 16.4 (9.3-23.5) |
| Tibet | 207/1157 | 9.8 (0.1-22.6) |  | 165/864 | 11.8 (0.1-26.7) |
